# Uncommon Collision Tumors: Dermoscopic and Histopathological Features of Basal Cell Carcinoma Overlying Dermatofibroma

**DOI:** 10.3390/dermatopathology12020010

**Published:** 2025-03-25

**Authors:** Amal Makansi, Charlotta Enerbäck, Maria Madentzoglou, Georgios Kravvas, Sandra Jerkovic Gulin

**Affiliations:** 1 Department of Dermatology, Linköping University Hospital, 581 85 Linköping, Sweden; 2 Department of Biomedical and Clinical Sciences, Faculty of Health Sciences, Linköping University, 581 83 Linköping, Sweden; 3 Department of Histopathology, Linköping University Hospital, 581 85 Linköping, Sweden; 4 Department of Dermatology, University College London Hospitals, 235 Euston Road, London NW1 2BU, UK; 5 Department of Dermatology and Venereology, Ryhov County Hospital, Sjukhusgatan, 553 05 Jönköping, Sweden; 6 Division of Cell Biology, Department of Biomedical and Clinical Sciences, Faculty of Medicine and Health Sciences, Linköping University, 581 83 Linköping, Sweden

**Keywords:** dermatofibroma, basal cell carcinoma, collision tumors, dermoscopy

## Abstract

Dermatofibromas (DFs) represent prevalent benign fibrohistiocytic tumors, typically manifesting as solitary lesions. In the majority of cases, the clinical presentation and dermoscopic and histopathological features of DFs adhere to a characteristic profile. However, DFs may exhibit atypical clinical presentations and, more commonly, histologic attributes, posing challenges in differential diagnosis. Both DFs and basal cell carcinomas (BCCs) are frequently encountered cutaneous lesions, each characterized by distinct clinical and dermoscopic features and microscopic morphology. The simultaneous occurrence of these two entities within the same lesion is rare. DFs have been documented to form collision tumors in conjunction with a spectrum of benign and malignant lesions, encompassing not only BCC but also balloon cell nevus, squamous cell carcinoma (SCC), and melanoma. Alterations in the epidermis overlaying a DF range from simple hyperplasia to the proliferation of basaloid cells. Accurate diagnosis, leading to the complete excision of the lesion, is contingent upon the recognition of dermoscopic criteria, precluding misinterpretation as a benign lesion. We present two cases of collision tumors comprising DF and BCC. This case report underscores the paramount importance of dermoscopy and adherence to dermoscopic criteria in the assessment of collision lesions and the diagnostic process related to cutaneous malignancies.

## 1. Introduction

Cutaneous collision tumors, while infrequent, have been comprehensively documented in the literature. Basal cell carcinomas (BCCs) have been described before simultaneously with a variety of one or more non neoplastic or neoplastic lesions [1,2,3,4]. Frequently reported combinations involve BCC in conjunction with nevi, BCC combined with seborrheic keratosis, and nevi coexisting with seborrheic keratosis [3,4,5,6,7,8,9]. The concurrent occurrence of BCC and dermatofibroma (DF) represents a rare phenomenon, with sparse documentation in the medical literature [2,3,8,10]. The comprehension of the underlying mechanisms and clinical and dermoscopic features and the development of appropriate management strategies for such instances are imperative for the provision of optimal patient care. DFs are one of the most prevalent, benign skin tumors and display a range of histopathological types. DFs exhibit a slight predilection for females and are frequently localized on the limbs. These fibrosing cutaneous lesions emerge within the dermis and occasionally extend into the subcutaneous tissue. The hypothesized etiology involves either neoplastic processes or a reactive response to trauma and arthropod bites [10,11]. Basal cell cancer is the most common malignant tumor. Epidermal changes overlying DF have been thoroughly described before, including the frequent basaloid proliferation overlying dermatofibroma, which is benign but can eventually cause diagnostic difficulty in differential diagnosis from BCC [12,13]. The discussion about the colocalization of DF and BCC often centers on the debate between the absence of malignant potential in reactive basaloid cell hyperplasia (BCH). Nevertheless, instances have been documented where true BCCs are observed to overlay DF, representing a rare scenario involving two common tumors. This specific scenario presents a challenge, as such cases of colocalization can be misinterpreted as BCH. It is crucial, therefore, to discern clinically relevant clues that facilitate the effective diagnosis of lesions, ensuring the accurate identification of true malignant BCCs that may masquerade as atypical DFs [2,3,11,12,13,14]. Epidermal changes overlying DF have been thoroughly described before, including the frequent basaloid proliferation overlying dermatofibroma, which is benign but can eventually cause diagnostic difficulty in differential diagnosis from BCC. Histologically, the frequent presence of mature adnexal structures in the context of basaloid proliferation overlying DF help in the distinction of malignant tumors including BCC, without the need of immunohistochemistry for diagnosis [15].

Here, we present two cases of collision tumors of BCC and DF. The cases were identified and retrieved from the medical documentation system at the Department of Dermatology, Linköping University Hospital, Sweden. Both cases underwent interdisciplinary evaluation, involving collaborative discussions among specialist dermatologists and cutaneous pathologists. Informed consent was obtained from the patients, explicitly authorizing the use of their clinical images and relevant medical information for inclusion in this case report.

## 2. Case 1

**Figure 1:**
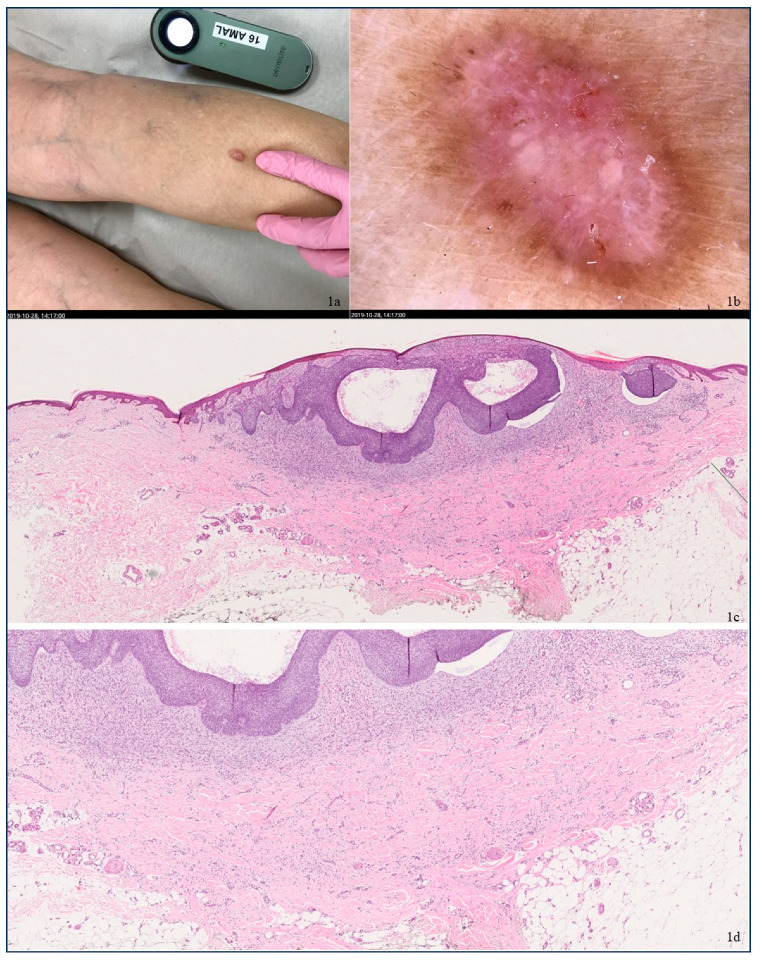
(**a**) A reddish nodule with a distinctive brownish border. (**b**) Dermoscopic examination revealed a white patch in the central area and a fine light brown pigmented network at the periphery, gray/blue pigmented blotches, arborizing vessels centrally, and microulceration. (**c,d**) Images showing 1c (×2)–1d (×10) magnification of histopathology. Stain: hematoxylin and eosin. The histopathological examination confirmed a collision lesion comprising a nodular basal cell carcinoma (BCC) and a dermatofibroma (DF). The basaloid tumor was connected to the epidermis and was nodular and well circumscribed within the dermis. It consisted of large basaloid nodules exhibiting peripheral nuclear palisading, mitotic activity, apoptosis, and central cyst formation. In the surrounding stroma, cleft formation between tumor lobules and stroma was observed, along with fibromyxoid stromal changes. Beneath the basaloid tumor, a dermal lesion composed of spindled fibroblasts or histiocytes was identified, with no cytologic atypia or increased mitotic activity. Additionally, focal multinucleated giant cells, eosinophilic collagen fibers, and scattered blood vessels were noted.

A 72-year-old female with no prior history of cutaneous malignancies presented with a gradually enlarging pigmented lesion on the right leg. The lesion measured 8 mm × 11 mm and appeared as a reddish nodule with a distinct brownish border and displayed the characteristic dimple sign of DF (Figure 1a). Dermoscopy revealed a central white patch and a fine, light brown pigmented network at the periphery, suggestive of DF (Figure 1b). However, the presence of atypical gray blue pigmented blotches, arborizing vessels centrally, and microulceration raised suspicions of BCC. Additionally, the patient was also found to harbor a second nodular BCC on the nose. Surgical excision of both lesions was undertaken. Histopathological examination of the right leg lesion confirmed the presence of fully excised nodular basaloid proliferation consistent with nodular BCC, Glas type IA according to Glas classification [16], overlying a DF. The basaloid mass was well demarcated and nodular and adhered to the epidermal surface, with a tumor front curving into the upper dermis. Surrounding stromal infiltration by small, slightly elongated cells was also noted (Figure 1c,d). Histopathological examination of the nose lesion found a fully excised morpheic BCC. The patient was placed under regular follow-up without any evidence of a recurrence at 3.5 years post-surgery.

## 3. Case 2

**Figure 2:**
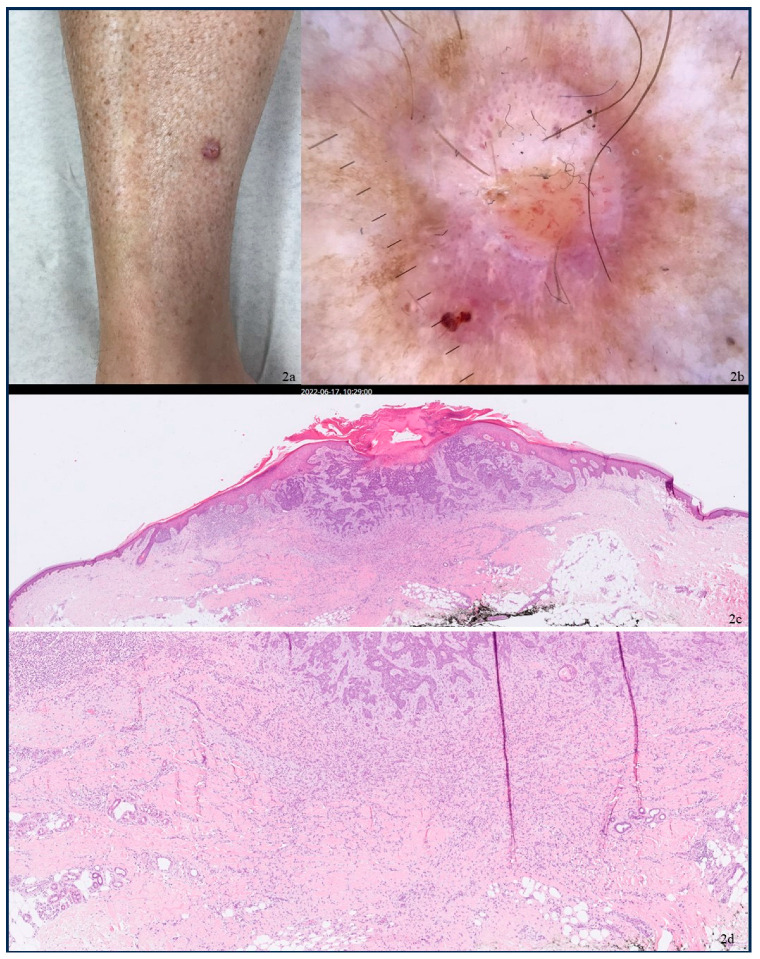
(**a**) Nodular red/brown lesion on the right shin with central ulceration. (**b**) Dermoscopic features: a central white structureless patch surrounded by a fine light brown reticulated network and microulceration. Glomerular vessels at the periphery, transitioning to loop vessels at the center. (**c**,**d**) Images showing c (×2)–2d (×10) magnification of histopathology. Stain: hematoxylin and eosin. The histopathological examination identified a collision lesion comprised of a dermatofibroma (DF) and an infiltrative basal cell carcinoma (BCC). The ulcerated basaloid tumor was connected to the epidermis and demonstrated infiltration into the dermis. The tumor consisted of irregular basaloid cell nests, exhibiting variability in peripheral nuclear palisading, mitotic figures, and apoptotic activity. Sparse cleft formation between tumor lobules and the surrounding stroma was observed, along with fibromyxoid stromal changes. Beneath the infiltrative basaloid tumor, a relatively well-circumscribed but unencapsulated lesion was identified, composed of spindled fibroblasts and histiocytes, lacking cytologic atypia or increased mitotic activity. Focal multinucleated giant cells, eosinophilic collagen fibers interspersed among fibroblasts, and scattered blood vessels were also present.

We present the case of a 61-year-old female who reported a longstanding lesion on the right shin. Although the lesion did not demonstrate progressive growth, it exhibited persistent non-healing characteristics, resembling a chronic wound. The patient had a background of benign seborrheic keratoses but not of skin malignancies. Upon clinical examination, a nodular red-brown lesion, measuring 9 mm in diameter, was identified on the right shin, characterized by central ulceration (Figure 2a). Dermoscopy revealed a central white structureless patch, surrounded by a fine, light brown reticulated network. Additionally, glomerular vessels were observed at the periphery, transitioning into loop vessels centrally (Figure 2b). A surgical excision was performed, and the histopathological analysis of the lesion confirmed a collision tumor, comprised of a DF and an infiltrative BCC Glas type II according to Glas classification (Figure 2c,d) [16]. At one year of follow-up, the patient exhibited no evidence of a recurrence.

## 4. Discussion

Dermoscopy is a valuable tool in differentiating dermatofibroma (DF) from basal cell carcinoma (BCC), aiding in clinical decision making. Dermoscopically, DFs typically exhibit a central white scar-like patch with a peripheral delicate pigment network, whereas BCCs are characterized by arborizing vessels, ovoid nests, and blue-gray globules. When features of both entities are observed within the same lesion, clinicians should consider the possibility of a collision tumor and strongly contemplate histopathological examination to confirm the diagnosis and guide appropriate management [1,7,8,17,18,19]. Recognizing these dermoscopic patterns enhances diagnostic accuracy and reduces the need for unnecessary biopsies. Collision tumors involving DF and BCC are rare, posing diagnostic challenges due to overlapping clinical and dermoscopic features.

The frequent benign basaloid proliferation overlying dermatofibroma is a diagnostic pitfall in rare cases of collision tumors consisting of DF and true BCC. Histologically differential diagnosis of DF, depending on the type of DF, includes lesions such as dermatofibrosarcoma protuberans, spindle cell melanoma, squamous cell carcinoma, superficial leiomyosarcoma, and even atypical fibroxanthoma/pleomorphic dermal sarcoma. Immunohistochemistry (IHC) helps in the diagnosis of difficult cases [10,20,21]. Differential diagnosis of BCC histologically includes benign basaloid proliferation overlying DF but even benign and malign adnexal tumors or other tumors such as Merkel cell carcinoma [21]. Collision skin lesions are diverse combined lesions of either benign and/or malignant lesions [12,13], which often have atypical presentation [7,9]. Among these, BCC has been reported in a few cases with DF [1,2,3,11,14,18]. However, BCC has been simultaneously described in the literature with variety of benign and malign lesions such as adnexal, epidermal, melanocytic, and vascular tumors [12,13].

In both cases presented, dermoscopy revealed atypical findings suggestive of both dermatofibroma (DF) and basal cell carcinoma (BCC), necessitating histopathological confirmation. Histologically, the primary differential diagnosis in both cases was benign basaloid proliferation overlying DF. However, certain features—including the presence of mitosis and apoptosis in basaloid nodules, cleft formation between tumor lobules and stroma, fibromyxoid stroma, absence of hair follicle differentiation and clear cells, and an infiltrative growth pattern in one case—do not support a diagnosis of benign basaloid proliferation overlying DF. All the above findings have been described as clues in the differential diagnosis of BCC versus benign basaloid proliferation overlying DF. Immunohistochemistry may also be used in difficult cases. Among immunohistochemistry stains examined, CK20-positive Merkel cells are present in benign basaloid proliferation overlying DF but absent in BCC. Ki67 and p53 as well as other stains have also been tested with variable results. In the cases presented above, no immunohistochemistry was performed. The diagnosis of a collision tumor involving basal cell carcinoma (BCC) and dermatofibroma (DF) was established based on histopathology, which confirmed true BCC overlying DF, rather than reactive basaloid cell hyperplasia (BCH). Accurate diagnosis is crucial to ensure complete surgical excision, particularly given the infiltrative potential of BCC. In both cases, excision with adequate margins resulted in successful tumor removal without evidence of recurrence. These findings underscore the importance of dermoscopy and histopathology in evaluating atypical lesions and highlight the need for further research on the pathogenesis and management of DF-BCC collision tumors.

## 5. Conclusions

This report underscores the complex histopathological interplay in collision tumors, specifically involving DF and infiltrative BCC. This study highlights the crucial role of dermoscopy and thorough clinical evaluation in identifying atypical features suggestive of malignancy. The successful excision outcomes emphasize the importance of considering BCCs in DFs with atypical characteristics, reinforcing excision as a definitive and effective treatment option. Documenting such cases contributes to refining diagnostic criteria and improving clinical management strategies for rare cutaneous collision tumors.

## Data Availability

The data presented in this study are available on request from the corresponding author. The data are not publicly available due to privacy and ethical restrictions.
